# Yeast Protein Extract Emulsions Supplemented with Polyphenolic Compounds: Physical, Chemical and Stability Properties of Colorful Emulsions

**DOI:** 10.3390/antiox15030351

**Published:** 2026-03-11

**Authors:** Bernardo Almeida, Ana Catarina Costa, Filipe Vinagre, Catarina Prista, Filipe Centeno, Victor de Freitas, Anabela Raymundo, Susana Soares

**Affiliations:** 1 REQUIMTE, LAQV, Rede de Química e Tecnologia, Laboratório Associado para a Química Verde, Department of Chemistry and Biochemistry, Faculty of Sciences, University of Porto, Rua do Campo Alegre, s/n, 4169-007 Porto, Portugal; bernardoalmeida811@hotmail.com (B.A.); vfreitas@fc.up.pt (V.d.F.); 2 LEAF—Linking Landscape, Environment, Agriculture and Food Research Center, Instituto Superior de Agronomia, University of Lisbon, Tapada da Ajuda, 1349-017 Lisbon, Portugal; acatarinacosta@isa.ulisboa.pt (A.C.C.); fafvinagre@gmail.com (F.V.); cprista@isa.ulisboa.pt (C.P.); anabraymundo@isa.ulisboa.pt (A.R.); 3 Associated Laboratory TERRA, Instituto Superior de Agronomia, University of Lisbon, Tapada da Ajuda, 1349-017 Lisbon, Portugal; 4 Proenol S.A., Travessa das Lages 267, 4410-308 Canelas, Portugal; filipe.centeno@proenol.com

**Keywords:** yeast protein extracts, yeast-derived emulsifiers, polyphenols, butterfly pea flower, red cabbage, anthocyanin-protein interactions, emulsion structuring, lipid oxidation stability

## Abstract

The growing demand for clean-label, plant-based foods is accelerating the development of vegan emulsified products that avoid synthetic additives while delivering appealing sensory and health-related attributes. We formulated naturally colored, mayonnaise-like oil-in-water emulsions using 55% canola oil and yeast protein extracts (YPEs) as emulsifiers and polyphenol-rich ingredients derived from red cabbage and butterfly pea flower. The resulting systems were characterized for rheological behavior, texture, droplet-size distribution, lipid oxidation (peroxide value) and microbiological stability. Two distinct YPEs produced emulsions with different microstructural and mechanical properties, highlighting the role of protein composition on emulsion architecture. Incorporation of anthocyanin-rich polyphenol matrices (red cabbage extracts characterized by predominantly simple acylations and butterfly pea flower extracts containing complex acylations, both at similar purities) modulated emulsion structuring and stability during storage, beyond color delivery. Overall, polyphenol addition strengthened emulsion structure, as evidenced by a significant increase in plateau modulus from 621 Pa to 1428 Pa in emulsions with complete YPE and butterfly pea extract and mitigated lipid oxidation, supporting their use as partial replacement options for additives such as EDTA in clean-label formulations. These findings provide a practical basis for designing functional, and visually attractive vegan emulsions that align with consumer demand for additive-reduced products.

## 1. Introduction

Formulating stable, appealing emulsions with clean-label additives and without animal-derived proteins remains a challenge in the development of plant-based food systems. The need for these alternative products is driven by the increasing consumer awareness of health, environmental, and ethical concerns, which continue to shape the current food market trends [1,2]. Beyond the substitution of animal-based proteins, market trends indicate that consumers want healthier food products, with reduced fat content, and less artificial and synthetic ingredients [3,4].

Mayonnaise is an oil-in-water emulsion widely used around the world as a sauce and as a base formulation for the study of emulsion science [5]. Its traditional composition uses oil, egg yolks, and an acidifier such as lemon juice or vinegar, but recent consumer trends have led the scientific community to look for alternatives to animal proteins [6]; indeed, industry analysis places the global vegan mayonnaise market at 1 billion USD with projected growth of ~9% CAGR this decade [7]. While plant-based reformulations are increasingly common, achieving the same technological, sensorial, and safety characteristics as conventional mayonnaise formulations remains technically challenging. Therefore, the central aim of this study was to elucidate how processing-dependent differences in yeast protein extract (YPE) functionality translate into structure–function relationships in high-oil oil-in-water emulsions. It was hypothesized that (i) differences in YPE composition and thermal transition profiles are associated with differences in emulsion microstructure and bulk rheology, and (ii) anthocyanin-rich polyphenol ingredients modulate these structure–function relationships via matrix- and anthocyanin-chemistry-dependent interactions with the continuous phase and/or the oil–water interface. Accordingly, two YPEs (complete and neutralized) were compared in mayonnaise-like emulsions with either whole polyphenol matrices or corresponding extracts to disentangle polyphenol-driven effects from broader matrix contributions.

The usage of yeast protein has come up as an alternative to traditional emulsifiers, as a cheap, widely available, and food-grade option [8,9]. Popular species include *Yarrowia lipolytica*, *Pichia jadinii*, *Kluyveromyces*, and *Saccharomyces cerevisiae*, among others [10]; in the present study *Saccharomyces cerevisiae* was used. Yeast protein extracts (YPEs) have the added benefit of being rich in glutamic acid [11], invoking the umami taste, improving the sensory profile of the final product. Additionally, the wine industry is a large provider of spent yeast, which could be used for the production of yeast protein extracts, further contributing to circular economy [12].

Polyphenols are naturally occurring compounds found in fruits, vegetables, and flowers that can act as antioxidants through radical scavenging [13], metal chelation [14], and, depending on their polarity, preferential localization at the oil–water interface [15]. They also exhibit antimicrobial activity via mechanisms such as membrane disruption [16] and enzyme inhibition, with effects that are often pH-dependent [17] and strongly influenced by the surrounding food matrix, so they should be considered potential contributors to (rather than direct substitutes for) conventional additives.

They also have well-documented anti-microbial effects [18,19], which make them ideal substitutes for the usual artificial additives. As a benchmark for oxidative-stability additives, calcium disodium EDTA is permitted at up to 75 ppm in mayonnaise in the United States [20] and up to 75 mg/kg in emulsified sauces in the European Union [21]. This regulatory context underscores the relevance of investigating formulation strategies that may help reduce additive use in these food systems.

In this context, the production of a vegan emulsion harnessing the potential of YPE and polyphenols introduces an innovative strategy to boost the functional and technological properties of the final product. Indeed, polyphenols have been extensively used in conjunction with proteins to modulate certain aspects, such as spatial arrangement [22], emulsifying power [23], and improve polyphenol antioxidant power [24].

Additionally, polyphenols present an opportunity to partially or totally replace industry-standard additives like EDTA [25], and potassium sorbate [26] by providing dual functionality, with anti-microbial and antioxidant activities properties.

In the present work, two different colorful polyphenolic sources (red cabbage and butterfly pea flower) and two different YPEs were introduced to produce vegan mayonnaise. YPEs were obtained by a confidential process, with the main difference being a neutralization step, removing most of the flavor and aroma inherent to yeasts. The neutralization procedure used to produce YPEn is proprietary and cannot be disclosed by the supplier. However, neutralization can alter protein functionality beyond aroma/color by changing pH and ionic conditions of protein exposure during processing, which can influence protein charge, solubility, aggregation, hydrophobic exposure, and thus interfacial adsorption and bulk rheology.

Red cabbage (*Brassica oleracea var. capitata f. rubra*) and butterfly pea flower (*Clitoria ternatea*) were applied either directly or after a prior extraction and purification to produce a polyphenol-enriched vegan formulation. To distinguish polyphenol-driven effects from broader ingredient matrix effects, both whole polyphenol matrices and their corresponding extracts were evaluated. Whole matrices contain additional constituents (e.g., polysaccharides, sugars, minerals, and organic acids) that can independently influence emulsion structuring and microbial outcomes, whereas the extracts provide a more polyphenol-enriched fraction. Comparing both formats therefore allows us to attribute observed changes more specifically to polyphenol chemistry versus co-ingredient matrix contributions.

The produced formulations were then tested for rheological, microbiological and antioxidant activity. Droplet size and microbial stability over-time studies were also conducted, to assess the developed product’s potential as a future marketable sauce.

## 2. Materials and Methods

Emulsions were prepared using ingredients kindly supplied by Casa Mendes Gonçalves (Golegã, Portugal), except for the yeast protein extracts, which were kindly supplied by ProEnol (ProEnol, Indústria Biotecnológica, SA, Porto, Portugal.). Two yeast protein extracts (YPEs) were used; for this, *Saccharomyces cerevisiae* cells were initially processed to remove cell walls [27], and a complete yeast protein extract (YPEc) was obtained. Further processing steps were employed to considerably reduce the intensity of aromas, flavors, and color, obtaining a neutral yeast protein extract (YPEn). The remaining reagents used were purchased from Sigma-Aldrich (Saint Louis, MO, USA). Whole plant sources were used: freeze-dried red cabbage powder (RCFD) and butterfly pea commercial extract (BPc) (produced by Zhejiang Binmei Biotechnology Co., Ltd., Taizhou, Zhejiang, China). Polyphenolic extracts were produced by solid–liquid extraction and characterized by LC-MS, as described in the Supplementary Information Figure S1 and Tables S1 and S2, resulting in red cabbage extract (RCExt) [28] and butterfly pea flower extract (BPExt) [28,29]. Protein content of each yeast protein extract (YPE) was determined by Kjeldahl method [30], also described in the Supplementary Information.

### 2.1. Emulsion Preparation

All emulsions were prepared following the same base formulation using a Thermomix TM6 (Vorwerk, Wuppertal, Germany). Firstly, YPEn and YPEc were added up to 1% (*w*/*v*) protein content (values optimized in an earlier work [9]), along with food powder or polyphenolic extracts, salt, modified starch, sugar, potassium sorbate, and EDTA dissolved in water. Then, emulsification was carried out by slowly adding canola oil up to a final concentration of 55%. Finally, vinegar was added and thoroughly mixed. For the polyphenolic-enriched emulsions, the previous step was performed with food powders, where 1.5 g·L^−1^ of food powder or polyphenolic extract was added to water, and the robot was set to max speed for 30 s. After this step, a similar protocol to the one described above was carried out. Following preparation, emulsions were kept in standardized glass containers at 4 °C, ensuring that all measurements were taken the following day to ensure complete emulsion stability.

### 2.2. Differential Scanning Microcalorimetry (DSC)

Protein conformation, conformational stability, and composition heterogeneity of the YPEs were assayed through differential scanning microcalorimetry [31] (µDSC) using a Setaram microDSC high-sensitivity calorimeter (Setaram, Caluire, France). For each scan, two cells were loaded with the sample and ultrapure milli-Q™ water as a reference. YPEs were dissolved in water in a final concentration of 2.5 and 1.5 mg·ml^−1^ for YPEn and YPEc, respectively, as determined by BCA colorimetric assay, to obtain a robust and comparable calorimetric signal under identical instrument settings. Accordingly, enthalpy values are not compared across YPEn and YPEc, and concentration differences are considered a limitation for any enthalpy-based interpretation. Two heating and cooling scans were run to study the reversibility of the transitions at a scanning rate of 1 °C·min^−1^, from 20 °C to 90 °C.

### 2.3. Texture Measurements

Texture Profile Analysis was carried out in a TA.XT Plus texturometer (Stable Micro Systems, Surrey, UK) with a 5 kg load cell. All measurements were taken in a temperature-controlled room (20 °C ± 1 °C), with each emulsion being analyzed a minimum of 5 times. Emulsions were placed in the same glass container (6 cm diameter, 4 cm depth) to ensure comparable results. A 19 mm diameter cylindrical probe was lowered 15 mm into the sample at a rate of 1 mm/s [32], with a pause time of 5 s. A “two bite test” analysis was conducted, a force vs. time graph was obtained, and firmness and adhesiveness were calculated. Firmness (N) was determined by the maximum force of the first compression cycle and represents the force necessary to compress the material; adhesiveness (N·s) corresponds to the negative area of the graph and describes the work necessary to remove the probe from the sample [33].

### 2.4. Rheology Measurements

Steady-state flow and linear viscosity measurements were carried out in a stress-controlled rheometer (Haake Mars III, Thermo Scientific, Waltham, MA, USA) equipped with a UTC Peltier for temperature control. Small amplitude oscillatory shear (SAOS) measurements were performed with a cone and plate system (35 mm diameter, 2°), within the previously assessed linear viscoelastic region (LVR assessed at a fixed frequency of 1 Hz, resulting in a strain amplitude of 0.07%). The mechanical spectrum was obtained and plotted as storage modulus (*G*′) and loss modulus (*G*″) vs. frequency. The plateau modulus, $G_{N}^{0}$, was estimated by the value of *G*″ corresponding to the minimum tanδ value, as described in the literature [34]. Steady-state flow curves were obtained with a serrated parallel system (35 mm diameter, with a 1.5 mm gap) within a shear rate range from 10^−7^ to 500 s^−1^. Viscosity vs. shear rate graphs were plotted and fitted against a Cross–Williamson model [35,36] (Equation (1)) using the Solver package for Microsoft Excel.

(1) $$\eta =\frac{\eta_{0}}{1+{\left(k\dot{\gamma }\right)}^{\left(1-n\right)}}$$

where *η*_0_ is the zero shear rate limiting viscosity at low shear rates (Pa∙s), *k* is the consistency coefficient (s), and *n* is a dimensionless shear-thinning index.

### 2.5. Droplet Size Distribution

Droplet size was determined using a Partica La960 V2 Laser Scattering Particle Size Analyser (Horiba, Kyoto, Japan) in wet mode, using a refractive index of 1.46 [37]. Emulsions were diluted and homogenized in deionized H_2_O before determination, at 20 °C, in triplicates [32]. Sauter diameter (*d*_3,2_) was calculated (Equation (2)), which expresses the mean diameter for most droplets, using LA-960, version V2-885 software:

(2) $$d_{\left(3,2\right)}=\frac{\sum n_{i}d_{i}^{3}}{\sum n_{i}d_{i}^{2}}$$

where *n_i_* is the number of droplets that have di diameter.

### 2.6. Color Analysis

Emulsion color was analyzed with a colorimeter (CR-400 Chroma Meter, Konica Minolta, Tokyo, Japan), using CIELab system, analyzing 5 replicates per sample. To determine the color variations between samples, ∆E* was calculated based on Equation (3):

(3) $$∆E=\sqrt[{2}]{{\left(L_{1}^{*}-L_{2}^{*}\right)}^{2}+{\left(a_{1}^{*}-a_{2}^{*}\right)}^{2}+{\left(b_{1}^{*}-b_{2}^{*}\right)}^{2}}$$

where L* indicates lightness, a* is the red/green coordinate, and b* is the yellow/blue coordinate.

### 2.7. pH Determination

Emulsion pH was determined with a pH meter (Seven Compact pH meter S220, Metter Toledo, Columbus, OH, USA). Five replicates were taken for each sample.

### 2.8. Oxidative Stability

Peroxide values (PVs) were determined by the official AOCS method CD8-53 [38]. Briefly, a small aliquot of each produced emulsion was frozen at −80 °C for 1 h. Afterwards, the sample was placed in a 20 °C ultrasonicating bath for 2 min and then centrifuged at 10,000 *g* for 15 min. After, 0.5 g of extracted oil was weighed and dissolved in 3:1 chloroform/glacial acetic acid solution. After 0.5 mL of saturated potassium iodide solution was added, the mixture was incubated in the dark for 1 min and titrated with 0.1 M sodium thiosulfate solution, using starch indicator. *PV* was calculated according to Equation (4):

(4) $$PV\;\left(mEq\cdot {kg}^{-1}\right)=\;\frac{\left(S-B\right)\times N\times 1000}{sample\;mass}$$

where *S* is the volume of titrant used for the sample, *B* is the volume of titrated used for the blank and *N* is the normality of the sodium thiosulfate solution. To ensure that non-oxidized oils were used, a *PV* determination was performed on the non-emulsified canola oil, which displayed a *PV* of 3.2 mEq·kg^−1^.

### 2.9. In Vitro Antimicrobial Activity of Polyphenolics-Enriched Emulsions

In vitro antimicrobial activity of the produced emulsions was tested by the drop test method [39]. Selected microorganisms displayed in Table 1 were chosen to represent the main spoilage and safety-relevant groups for mayonnaise-like, acidic oil-in-water emulsions. The selected LAB (*Levilactobacillus brevis*, *Lacticaseibacillus rhamnosus*, and *Pediococcus pentosaceus*) are acid-tolerant spoilers commonly found in refrigerated or mildly acidic foods, while the yeasts (*Meyerozyma guilliermondii*, *Debaryomyces hansenii*, and *Zygosaccharomyces parabailii*) reflect key yeast spoilers of acidic, high-solids sauces, particularly *Zygosaccharomyces*, which is highly acid tolerant and frequently linked to spoilage of preserved acidic products. In addition, pathogenic species such as *Listeria innocua*, a common contaminant in refrigerated ready-to-eat goods, and *Salmonella typhimurium*, a common pathogen in egg-containing foods were also used.

After emulsion preparation, 1 g was transferred to a sealed tube and inoculated with 100 µL of each species described in Table 1, totaling 10^9^ CFUs per emulsion, per species [46,47,48,49,50,51]. Immediately after inoculation and at every time point (0, 7, 14, and 30 days), 100 mg of each inoculated emulsion was dissolved in 900 µL of ¼ Ringer solution (1:10) (2.25 g/L NaCl, 0.105 g/L KCl, 0.065 g/L CaCl_2_·H_2_O, and 0.05 g/L NaHCO_3_), and 10-fold dilutions were performed in a 96-well microplate, up to 10^−5^. Each dilution was then plated in the appropriate agar-containing media (Table 1) with a 3 µL droplet. After plating, lactic acid bacteria and yeast plates were incubated at 25 °C and pathogenic species at 37 °C for a minimum of 48 h before photographing. Every condition was plated in duplicates.

### 2.10. Statistical Analysis

All techniques described in this section were performed in triplicates and results were analyzed with ANOVA and Tukey’s HSD post hoc in GraphPad Prism v10.3.0 (GraphPad Software Inc., Boston, MA, USA), unless stated otherwise.

## 3. Results and Discussion

### 3.1. Texture Profile Analysis

**Figure 1:**
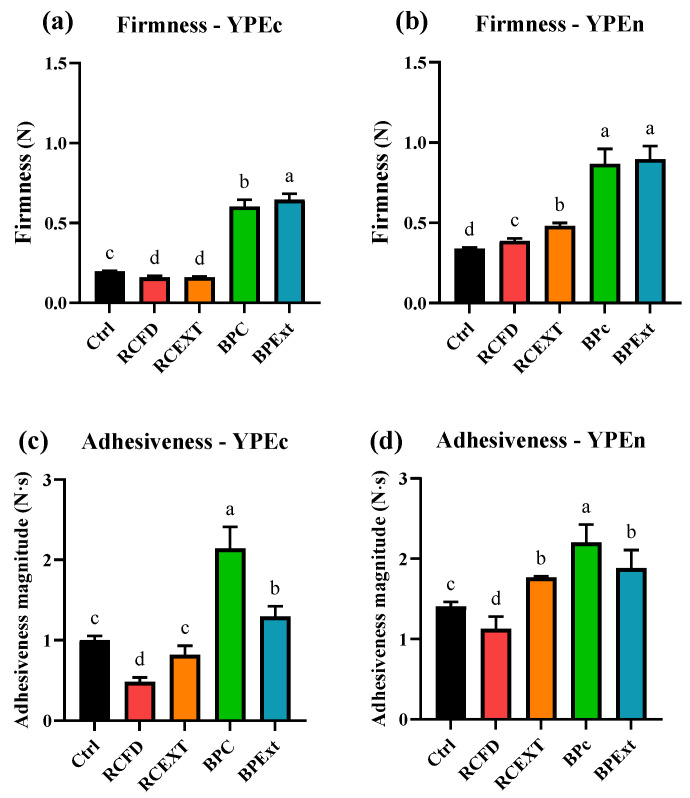
Firmness (N) (complete YPE (**a**) and neutral YPE (**b**)), and adhesiveness magnitude (N∙s,) (complete YPE (**c**) and neutral YPE (**d**)) values obtained from Texture Profile Analysis (TPA) for all types of emulsions produced. Different letters indicate statistical differences according to Tukey’s HSD Test (*p* < 0.05).

Texture measurements were conducted in the produced emulsions with complete (YPEc) and neutral YPE (YPEn), with either food powders or polyphenol extracts (Figure 1); control emulsions were made without any polyphenolic additives.

TPA showed that emulsions prepared with the two YPEs differed in texture parameters (Figure 1). Overall, emulsions made with YPEn exhibited higher firmness and higher adhesiveness than those made with YPEc. In addition, texture responses varied depending on the additive format, with different trends observed for whole food powders compared with the corresponding polyphenol extracts. This effect of whole food powders vs. polyphenol extracts have been previously reported [9].

In terms of firmness values, both YPEs produced statistically different control emulsions (statistically different firmness values: YPEc Ctrl = 0.199 ± 0.002 N, YPEn Ctrl = 0.340 ± 0.01 N, *t*-test, *p* < 0.001). Emulsions produced with YPEc showed a slight reduction in firmness when produced with both red cabbage matrices, whereas there was a very significant increase in firmness when butterfly pea matrices were used, with BPExt resulting in the highest firmness value. For YPEn emulsions, both polyphenol matrices led to an increase in firmness; RCExt displayed higher firmness than RCFD, whereas there was no difference between the two butterfly pea matrices. This could hint at significant differences in structural conformation of both YPEs used. Interestingly, this observation is also in line with previous data showing that protein partial denaturation could increase the interaction with anthocyanins [52].

Regarding adhesiveness, both YPEs produced statistically different control emulsions (statistically different adhesiveness values: YPEc Ctrl = −1.004 ± 0.050 N·s, YPEn Ctrl = −1.407 ± 0.053 N·s, *t*-test, *p* < 0.001). Different behavior between food powder and pure extract was the same for both types of emulsion—all matrices except for RCFD caused an increase in adhesiveness, with BPc resulting in the highest increase in this parameter.

**Figure 2:**
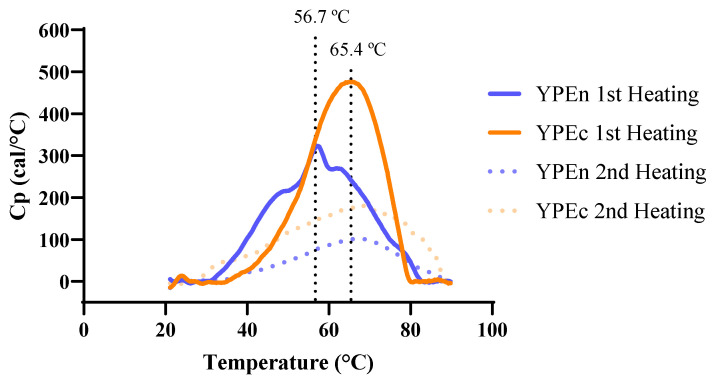
Differential scanning calorimetry of yeast protein extracts (YPEn in blue, YPEc in orange); colored dashed lines correspond to the 2nd heating cycle, and black dashed lines correspond to each sample’s Tm.

To verify the protein structure impact on the texture parameters, we performed differential scanning microcalorimetry, to infer the structure and conformation of the proteins present, as these are crucial characteristics for emulsion characterization, which is seen in Figure 2.

As protein denaturation peaks were being assayed, two heating cycles were applied to the samples to determine which phenomena were irreversible. As mentioned in the Methods section, protein extracts show different soluble protein concentrations, but concentrations were not standardized as the objective of the µDSC analysis was purely to compare thermal transition profiles. As such, peak temperatures and transition shapes were interpreted qualitatively. As shown in Figure 2, YPEc exhibits a narrower thermogram than YPEn, suggesting a less heterogeneous distribution of thermal transitions within the protein population. YPEc also shows a higher Tm, which is consistent with greater thermal conformational stability of at least part of the extract; this does not by itself demonstrate superior interfacial performance, but can be interpreted as an indirect indicator of stability rather than functional efficacy [53]. A higher degree of structuring could be a hindrance to interact with other compounds, such as polyphenols, and could prove less efficient to interact at the oil–water surface of oil droplets in an emulsion. YPEn displays a rather broad thermogram, implying a heterogeneous protein composition and a smaller T_m_, which could make it more favorable for emulsion structuring, as a less structured protein could have more hydrophobic groups exposed, necessary for emulsion stability [54].

Comparing the obtained parameters with the previously reported parameters by Ribeiro et al. for a commercial control [9], YPEc and YPEn produced with both butterfly pea matrices resulted in approximately similar firmness values to the commercial mayonnaise. Furthermore, YPEc with BPc displayed similar adhesiveness values to the reported commercial control whereas YPEn displayed similarity to the same control with RCExt, BPc and BPExt.

### 3.2. Rheological Behavior

**Figure 3:**
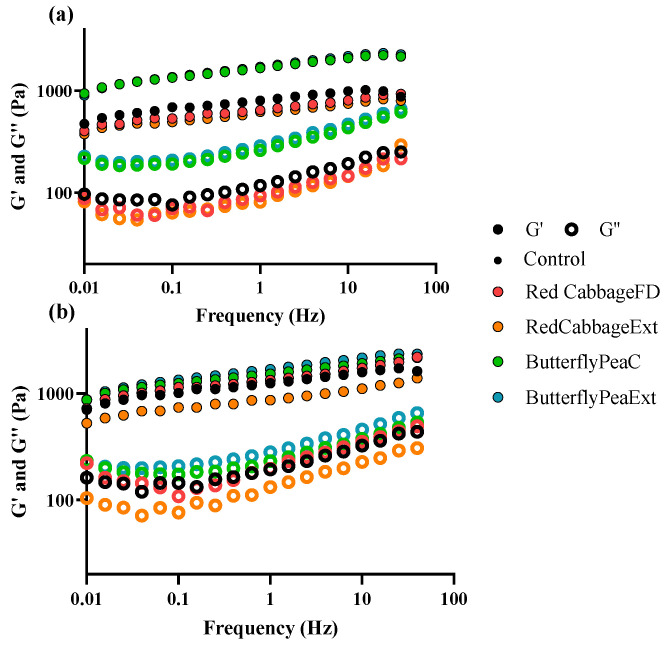
Mechanical spectra of emulsions produced with (**a**) YPEc and (**b**) YPEn where *G*′ is the storage modulus and *G*″ corresponds to the loss modulus.

Mechanical spectra obtained for all the produced emulsions are displayed in Figure 3, and values for moduli *G*′ and *G*″ at 1 Hz, as well as the estimated plateau modulus, are displayed in Table 2 and Table 3. 

Presented emulsions have higher *G*′ values compared to *G*″, with the YPEn ones (Figure 3b) having the highest dependency on frequency. Furthermore, *G*′ remained consistently higher than *G*″ by approximately one logarithmic unit across the frequency range, with both moduli exhibiting frequency dependency, commonly associated with weak-gel like behavior in food emulsions, as previously stated by Nunes et al. [55]. Comparing only the control emulsions between the two different types of YPE used, it is possible to conclude by the shape and slope of the obtained spectra that there are slight differences in the structuring of the emulsions [56]. Figure 2 shows that the two YPEs have different thermal transition profiles, which suggests differences in conformational stability and overall heterogeneity. In the emulsions, the YPEn control samples showed higher viscoelastic moduli than the YPEc controls (Table 2 and Table 3), indicating a more structured system and/or stronger droplet–droplet interactions under our test conditions. These bulk rheology differences are consistent with the idea that the extracts may differ in how they adsorb and build an interfacial film around droplets, but interfacial adsorption or composition were not measured directly. We therefore present this connection as a plausible explanation based on the rheological data, rather than a confirmed mechanism, and note that it should be verified with dedicated interfacial measurements.

Regarding YPEc emulsions (Table 2), the addition of both red cabbage matrices lowers all rheological parameters, albeit not significantly, indicating a possible destabilization of the structure network [57]. However, butterfly pea matrices exhibited completely opposite behavior, with values not only significantly higher than the control but also exceeding those of neutral emulsions for the same additive while achieving a very similar structuring.

The addition of butterfly pea, and consequently the formation of ternatin-coated droplets [58], could be compensating the unevenness of the protein-mesh around oil droplets, resulting in a far superior structuring of the emulsion due to the hydrogen bonds between droplets and the aqueous phase; furthermore, as studied by Kong et al. [59], although with a different acylation degree, acylation causes a significant increase in the lipophilicity of anthocyanins, which also further strengthens the protein-anthocyanin membrane surrounding the fat droplets. Although ternatin–protein interactions have not yet been reported, interactions between proteins and other anthocyanins are well documented, and their nature depends on both the protein and anthocyanin structures.

YPEc emulsions when made with butterfly pea matrices displayed *G*′ at 1 Hz and plateau modulus values much higher than the values displayed for commercial mayonnaise according to Ribeiro et al. [9] and similar values for control and red cabbage matrices.

As for YPEn emulsions (Table 2), the addition of RCExt causes a decrease in *G*′, *G*″ and plateau modulus values, while both butterfly pea matrices caused an increase in these values when compared to control. As demonstrated by previous studies, molecules that are able to establish hydrophobic interactions with proteins can potentially displace them from the oil/water interface, causing structure collapse [60,61]. Phenolic compounds have been shown to localize at protein-stabilized oil–water interfaces and, depending on their polarity and interactions, may co-adsorb with or partially displace interfacial proteins, which can modify interfacial film integrity and, in turn, emulsion structure. So, here it is hypothesized that the anthocyanins present in RCExt can potentially displace the proteins from the oil/water interface, leading to a destructuring of the emulsion [62,63]. Due to a much smaller concentration of polyphenol present in RCFD, this effect is not as pronounced so all rheological parameters are higher than control and RCExt. As YPEc did not exhibit the same behavior, this phenomenon is protein-size and configuration-dependent [64].

The obtained mechanical spectra of these emulsions suggested a small structural change in butterfly pea neutral emulsions, compared to control, which could be attributed to the highly complex acylated structure of the butterfly pea anthocyanins [28]. The presence of ternatins would introduce a large amount of hydroxyl groups, available for interaction with the protein, increasing its rigidity and therefore increasing emulsion structuring [65]. A secondary phenomenon could also be occurring where the ternatins stay at the oil–water interface, the polyphenolic backbone of the ternatins interact with the proteins and the acylated chains form a more cohesive layer around every droplet [66]; this would both create hydrogen bonds with the aqueous solvent, as well as hydrogen bonds and π-π stacking with other ternatins-coated droplets, strengthening the emulsion.

Finally, YPEn emulsions displayed higher *G*′ at 1 Hz and plateau modulus values than the reported commercial mayonnaise [9], for most conditions, except for RCExt for the reasons stated above.

### 3.3. Flow Behavior

**Figure 4:**
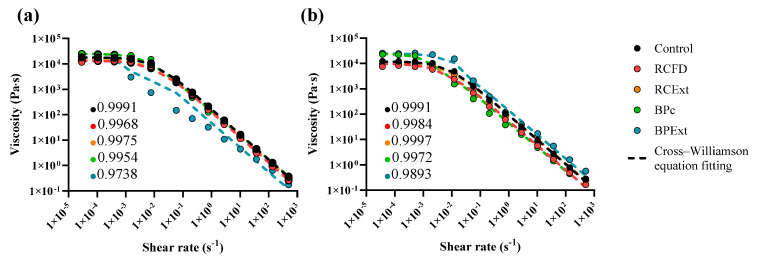
Flow curve of YPEc (**a**) and YPEn (**b**) emulsions with red cabbage powder (RCFD) and butterfly pea commercial extract (BPc) and corresponding polyphenol extracts (RCExt and BPExt, respectively). Dotted lines represent the fitted curve, according to the modified Cross–Williamson model (Equation (1)).

Flow curves for all the produced emulsions are displayed in Figure 4. All emulsions produced exhibit the typical shear-thinning behavior of a flocculated emulsion: a constant high viscosity region—first the initial Newtonian plateau, where the zero-shear viscosity (*η*_0_) can be determined, followed by the shear-thinning zone, where the apparent viscosity declines as the shear rate increases. An apparent yield stress of approximately 50–100 Pa was estimated from the onset of viscosity decay in the steady shear curves, reflecting the stress required to disrupt the flocculated network and initiate continuous flow. This behavior could be explained by the floccules’ interconnected network remaining intact under low stress, resulting in a high viscosity. When the applied shear rate is high enough, this network begins to disrupt, resulting in a decreased viscosity [67].

YPEn emulsions (Table 3) present overall higher zero-shear rate limiting viscosity values compared to YPEc; this could be due to the presence of a more heterogeneous protein conformation, as inferred by the DSC results, (Figure 2), which could result in a less tightly packed structure, as previously discussed. The zero-shear rate limiting viscosity values presented in Table 3 show that the highest undisrupted viscosity is due to the addition of the butterfly pea matrices, both commercial and purified extract. These results are aligned with the results displayed in Table 2 and Table 3, where the plateau modulus values are also higher for these matrices; this further confirms that the predicted $G_{N}^{0}$ suggests a more consistent emulsion structure [57]. These results are also concordant with the obtained firmness values displayed in Figure 1 and with the thermograms present in Figure 2. This effect is more accentuated in the case of the complete emulsions with pure BPF extract, where the *η*_0_ values are similar to the displayed values for the neutral YPE emulsions.

### 3.4. Droplet Size Stability over Time

**Figure 5:**
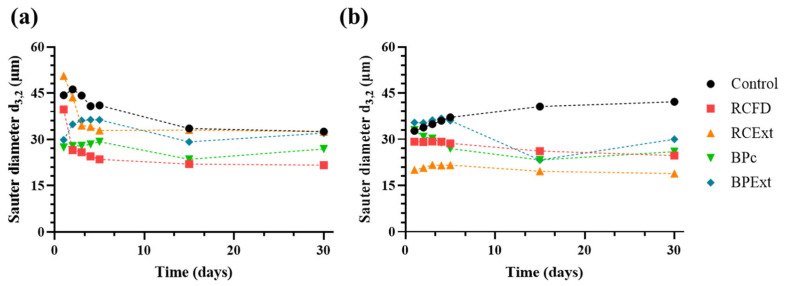
Sauter diameter (*d*_3,2_) over 30 days for (**a**) YPEc and (**b**) YPEn emulsions made with freeze-dried red cabbage (RCFD), red cabbage extract (RCExt), butterfly pea commercial extract (BPc), and butterfly pea pure extract (BPExt).

Droplet size monitoring is a crucial measurement for emulsion stability over time [68]. As such, emulsion stability was studied through particle size distribution measurements over a period of 30 days, displayed in Figure 5.

The natural tendency for suspended droplets is to aggregate/coalesce, forming larger droplets, increasing their diameter, and ultimately resulting in phase separation [69]. All emulsions exhibited relative stability throughout the monitored period, as seen by the comparable Sauter diameters at 15 and 30 days. However, smaller Sauter diameters were registered for all non-control YPEc emulsions, as well as for RCFD, BPc, and BPExt in YPEn, at the same time points.

In the case of RCFD and RCExt YPEn emulsions (Figure 5a), it is possible to see that during the first few days, the particle size decreases quite drastically—this may be due to the flocculation of smaller droplets. This trend may reflect reversible flocculation and measurement/sample-preparation effects, rather than a true reduction in primary droplet size. Laser diffraction reports an equivalent spherical diameter and cannot distinguish scattering from individual droplets versus droplet clusters; consequently, flocculated droplets may be recorded as a single, larger “apparent” particle. If such clusters are progressively disrupted during sampling, dilution, and/or circulation in the measurement cell, the apparent mean size can decrease even when the underlying droplet size remains unchanged. Similar limitations of laser-diffraction sizing in the presence of droplet clustering and dilution-related artifacts have been reported in the literature [70].

As time passes, the particles begin to repel each other, resulting in the final, stable drop size. Nonetheless, the particles produced are much larger than what is normally reported for oil-in-water emulsions [71,72], which could be attributed to the adjustments required to produce these emulsions: all formulations were produced at a laboratory scale, which likely resulted in a lower shearing power used, and consequently, the particles produced were larger than expected.

In practice, industrial rotor–stator systems and, in particular, high-pressure homogenization can deliver substantially higher and more reproducible energy densities, which would be expected to reduce the mean droplet size and narrow the size distribution, thereby increasing the interfacial area available for adsorption. As a consequence, key outcomes reported in this study, including interfacial film formation, rheological properties, and oxidation kinetics, may shift under industrial conditions. These scale-up considerations should therefore be taken into account when extrapolating the present results, and future work should first optimize processing conditions representative of commercial mayonnaise manufacturing.

In both emulsion types, adding BPExt led to a larger Sauter diameter over the first 5 days. This change likely reflects droplets coming together into loose clusters (flocculation) rather than pointing straight away to a single, specific molecular mechanism. In practice, the “growth” can simply come from non-specific aggregation: when steric or electrostatic repulsion is weakened and the droplet surfaces become a bit stickier, droplets form clusters that look larger hydrodynamically even if they have not actually coalesced. In the BPExt system, we suggest this general clustering tendency may be further reinforced by π–π interactions. The high degree of acylation likely increases the presence and exposure of aromatic groups at the interface, allowing more directional π–π stacking that helps stabilize droplet–droplet contacts [73].

### 3.5. pH and CIELab Measurements

Table 4 displays the CIELab measurements taken from each sample. ∆*E* values for control samples were calculated by comparing against the commercial control with the following CIELab values: L* = 92.55 ± 1.14, a* = −2.49 ± 0.27, and b* = 20.15 ± 0.79 [9]; remaining ∆*E* values were calculated by comparing each sample to its respective control.

The commercial control presents an overall brighter color as seen by the L* parameter in Table 4 when compared to both controls, with the neutral YPE control being the brightest produced emulsion; the produced emulsions also had lower b* values (which represent the yellow color). The differences between the commercial and the produced emulsions could be attributed to the lack of eggs, the higher oil content (65% *w*/*v* in commercial vs. 55% *w*/*v* in YPE emulsions), and the smaller drop size [74]. As expected from the images presented in Table 5, emulsions produced with pure polyphenolic extracts resulted in the greatest ∆*E*. However, it was possible to see the influence of the base color of each YPE on the final exhibited ∆*E*, as YPEn displayed greater ∆*E* values for every produced emulsion. As the production process of neutral YPE also removes inherent color tones, no sub-tone interference occurred, resulting in a more pronounced color change. It is important to refer that pH differences are likely to induce changes in the color of the emulsions, because anthocyanins are strongly pH-responsive pigments. In more acidic systems, anthocyanins are enriched in the flavylium cation form, which generally gives a stronger red/pink hue and improved color stability. As pH increases toward neutral, the equilibrium shifts toward quinoidal base and hydrated/hemiketal (and chalcone) forms, which can appear more bluish/purple but are often less intensely colored and less stable, leading to changes in lightness and overall Δ*E*. In general, Δ*E* ≈ 2–3 is considered close to a just-noticeable color difference for many observers, whereas Δ*E* > 5 is typically perceived as a clear and obvious difference. Accordingly, the Δ*E* values observed here suggest that all emulsions would present a readily noticeable color shift in comparison to respective control emulsions.

### 3.6. Oxidative Stability

**Figure 6:**
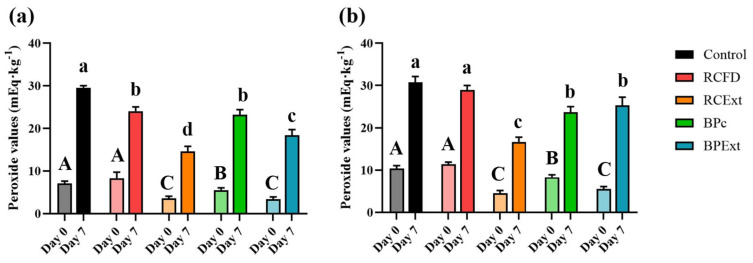
Peroxide values (mEq·kg^−1^) of (**a**) YPEc and (**b**) YPEn emulsions without EDTA after 7 days of storage at room temperature. Different letters above each column represent statistical differences according to Tukey’s HSD test (*p* > 0.05); day 0 (capital letters) and day 7 (lowercase letters) data were compared separately.

Since polyphenols are widely known antioxidants, this study was also focused on determining their ability to prevent emulsions oxidation and replace commonly used food additives with antioxidant capacity. In this way, for this study, all emulsions were produced without EDTA, the worst-case scenario, stored at room temperature without protection from light and the peroxide values (PVs) were determined at 0- and 7-day timepoints (Figure 6). This was done to accelerate oxidation and ensure that all oxidation differences from control were due only to the presence of polyphenol.

As indicated in the Materials and Methods section, the same PV determination was conducted on the oil used for the emulsions, which exhibited a value of 3.2 mEq·kg^−1^; as the control emulsions both exhibited higher PVs than the non-emulsified oil, this proves that the emulsification process itself causes some degree of lipid oxidation, as previously studied by Cengiz et al. [75]. Control emulsions produced with YPEc (Figure 6a) result in a lower PV when compared to YPEn (Figure 6b). The addition of RCFD did not result in a significantly different PV in either emulsion type upon production, while the addition of pure polyphenolic extract resulted in significantly lower PV values, as expected. After 7 days, all food powders and polyphenol extracts in YPEc emulsions resulted in a significantly lower PV compared to the control; in neutral emulsions, RCFD did not result in a significantly lower PV. Although the resulting PV was higher than initially expected, given the antioxidant properties often attributed to polyphenols [76], the added extracts were still able to slow down the oxidation of the produced emulsions even without the presence of EDTA, and exposure to room temperature and oxygen.

To frame these PV into context, the Codex Standard for Named Vegetable Oils (CXS 210-1999) sets maximum PV specifications of ≤10 mEq O_2_/kg for refined oils and ≤15 mEq O_2_/kg for cold-pressed/virgin oils (as marketed). Although PV in this study was measured on oil extracted from emulsions after accelerated storage (rather than on neat commercial oil), comparing the magnitude to these widely used specifications helps interpret the extent of primary oxidation. Under our test conditions, PV values ranged from 13 to 30 mEq O_2_/kg after 7 days, with none of the emulsions remaining within the refined-oil specification and indicating oxidation levels that would typically exceed refined-oil quality limits. So, this seems to indicate that the polyphenol extracts studied contribute to oxidative stability under accelerated, EDTA-free conditions and can support partial EDTA-reduction potential.

Previous studies have shown that polyacylated anthocyanins can exhibit lower in vitro antioxidant capacity in common aqueous assays (which primarily reflect bulk-phase radical scavenging and reducing power). However, their increased lipophilicity can enhance effectiveness in fat-rich foods and emulsions by promoting partitioning toward the lipid phase and/or the oil–water interface, where oxidation is initiated and propagated. In this way, a compound may appear weaker in aqueous in vitro tests yet provide stronger lipid-phase protection due to better localization at the main oxidation loci rather than higher intrinsic radical-scavenging activity [59,77]. In agreement with Klinger et al., even though ternatins present in butterfly pea display lower antioxidation values in in vitro assays [77], the complex acylations provide increased lipophilicity and over-time stability, increasing these compounds suitability for application in lipid-rich food products.

The behavior of BPExt, however, is different in the two types of emulsion: after 7 days, in YPEN emulsions the BPExt PV is not statistically different from BPc, whereas in YPEc emulsions, it was significantly lower than control and BPc. This result can be explained by different interactions between YPEn, YPEc, and butterfly pea flower polyphenols. Other authors have reported a negative impact on antioxidant capacity caused by protein interaction [78] potentially occupying -OH groups that are fundamental for radical scavenging. As YPEn displays a less structured protein conformation, Figure 2, this could be exposing more groups prone to interaction with the polyphenol [52], potentially lowering its antioxidant properties.

### 3.7. Antimicrobial Activity

**Figure 7:**
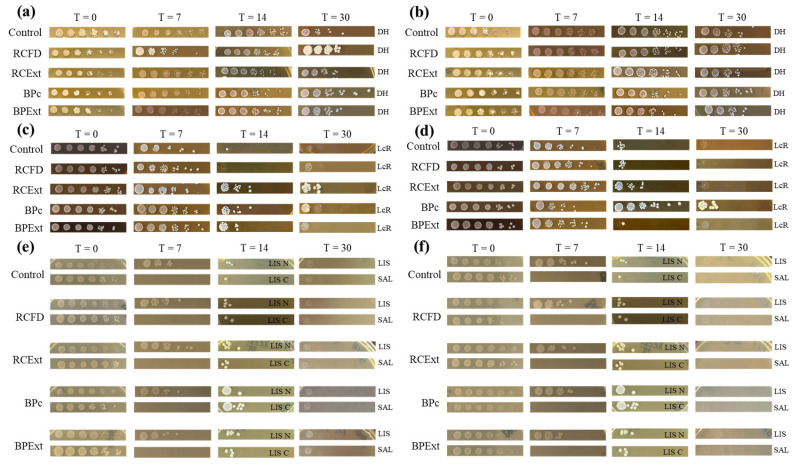
Plated 10-fold dilutions of complete (**a**,**c**,**e**) and neutral (**b**,**d**,**f**) emulsions inoculated with DH—*Debaryomyces hansenii*, LcR—*Lacticaseibacillus rhamnosus* and pathogenic bacteria (LIS—*Listeria innocua* and SAL—*Salmonella typhimurium*). Each cut-out represents a registered timepoint; one example of each replica is shown.

All the produced emulsions were inoculated with several microorganisms that are both reported to metabolize polyphenolic compounds (Table 1) and are common contaminants in the sauce industry. Figure 7 displays the yeasts, lactic acid bacteria, and pathogenic bacteria viability in YPE emulsions obtained at each registered time point.

Tested yeasts, Figure 7a,b and Figure S2a,b, survived for the 30 days of the experiment, with reductions of two logarithmic units, showcasing how resistant they are to both the polyphenolic additives and the potassium sorbate usually added to prevent microorganism growth [79]. After 14 days of exposure, a reduction in the number of CFUs of MG was observed of three logarithmic units; however, for the 30-day plates, the CFU numbers were similar to those at the start of the trial, possibly due to the adaptation of the cells to the imposed stress. While most additives did not result in a growth increase, BPc emulsions exhibited more CFUs by the end of the trial for all tested species. This result can be explained by the production process of the BPc—this extract is obtained by spray-drying using maltodextrin as an encapsulant, which the yeasts can readily feed on.

Lactic acid bacteria Figure 7c,d and Figure S2c,d growth appears to have greatly diminished, with registered reductions of six logarithmic units, after 14 days in control emulsions made with neutral and complete YPE, both for control, RCFD, and BPExt emulsions, not presenting any colonies by the 30-day mark. However, it is possible to observe that for both types of emulsions made with RCExt, some colonies of LB and LcR were still present at the 30-day mark; this is odd, as the expected behavior is that the purified extract would be more effective at inhibiting growth than the food powder/commercial extract, as showcased by the BPc and BPExt emulsions. However, both *Levilactobacillus brevis* and *Lacticaseibacillus rhamnosus* can metabolize anthocyanins similar to those present in black carrot juice and goji juice, and use them as a food source [80,81]; as RCFD has insufficient polyphenol content to serve as a food source, the selected LAB are unable to grow. Similarly to what happens on yeast-inoculated emulsions, BPc allows growth at the 30th day mark due to the presence of maltodextrin; however, as the anthocyanins present in butterfly pea are markedly structurally different, LAB cannot utilize them as a substrate.

A key limitation to keep in mind is that some LAB can actually use or transform anthocyanins and other polyphenols. As these compounds are broken down over time, their antimicrobial effect may weaken, especially in products where LAB are the main spoilage group. So, inhibition is detected early on; polyphenol-based “preservation” may not be equally durable in the presence of active LAB, highlighting the need to confirm these effects over longer storage and under more realistic mixed-culture conditions.

Regarding pathogenic bacteria (Figure 7e,f), the presence of the extracts decreased UFCs of both *Salmonella typhimurium* and *Listeria innocua*. This effect was more substantial for *Salmonella typhimurium*, with no colonies detected after 7 days in any of the produced emulsions, while for *Listeria innocua*, the complete inhibition was only observed by the 30th day. Importantly, this inhibition cannot be attributed to polyphenols alone because these mayonnaise-like systems also impose multiple antimicrobial hurdles (acidic pH, the presence of potassium sorbate in the base formulation, and refrigerated storage). We therefore interpret the observed reductions as the result of a combined-hurdle effect, where polyphenols likely contribute additional stress on top of the existing hurdles, rather than as evidence of polyphenols acting as a stand-alone preservative. Similar enhanced inhibition of *Salmonella typhimurium* under multi-hurdle conditions has been reported in other food matrices, including systems with lower-potassium sorbate levels [82].

## 4. Conclusions

In this study, vegan emulsions were prepared using food powders and polyphenol extracts derived from them, and their rheological, textural, oxidative, and microbiological stability parameters were evaluated. Emulsions exhibiting a range of visually distinct and attractive colors were successfully produced. The use of different YPEs led to variations in emulsion structuring, reflected in the mechanical profile and viscosity profiles. Moreover, anthocyanins with similar chemical structures but different degrees of acylation enabled a novel study into how acylation affects emulsion structuring and stability.

The two central hypotheses were: first, that processing-related differences between YPEs would translate into clear differences in emulsion structure and texture; and second, that anthocyanin-rich ingredients would further tune these properties depending on how they were added (whole matrix vs. extract) and on anthocyanin chemistry (including acylation). For that, vegan emulsions were prepared using food powders and polyphenol extracts derived from them, and their rheological, textural, oxidative, and microbiological stability parameters were evaluated. Overall, the results support the first hypothesis, since changing the YPE consistently led to differences in viscosity and mechanical/TPA profiles across the emulsions. The second hypothesis is partially supported: anthocyanin-containing ingredients influenced rheology, color, and short-term oxidation outcomes, and the microbial effects depended on the specific organisms and test conditions used. 

Overall, the addition of butterfly pea matrices improved emulsion structuring, producing a YPEc emulsion with rheological properties comparable to YPEn emulsions and to commercially available mayonnaise produced in a similar manner. Overall particle stability was maintained for the short period of time tested, although YPEn emulsions showed a reduction in particle size over 30 days when polyphenols were added. Oxidative stability trials also revealed that the addition of polyphenols resulted in a decrease in peroxide values even without the addition of EDTA; furthermore, butterfly pea extract, even with inferior TPC, resulted in comparable PV results. Polyphenols also exhibited a modest protective effect against lactic acid bacteria, without promoting the growth of yeasts and pathogenic bacteria compared to the controls. Taken together, the data confirm YPE-dependent structure–function differences and point to a formulation-dependent contribution of anthocyanin-rich ingredients, while also highlighting the need for direct interfacial measurements and longer-term shelf-life validation.

At the end, considering the application of these yeast protein extract-stabilized emulsions, they seem most suited to refrigerated vegan mayonnaise-style products and emulsified dressings, where polyphenol-rich ingredients can serve as natural colorants while providing partial oxidative protection. The approach may be particularly relevant for formulations aiming to reduce (rather than eliminate) chelating antioxidants such as EDTA, especially when combined with established hurdles such as low pH, appropriate packaging (oxygen/light limitation), and conventional preservatives at reduced levels. In addition, the visually distinct colors achieved here suggest opportunities for premium or niche products (e.g., colored sandwich spreads or specialty sauces) where appearance is a key differentiator, while texture can be tuned through the choice of YPE and polyphenol format.

## Figures and Tables

**Table 1:** Groupings, growth media, and studies that report polyphenolic compound metabolism for each microorganism used.

Group	Microorganism	Collection Number	Growth Media	Reported Interaction
Lactic acid bacteria	*Levilactobacillus* *brevis*	DSM 20054	De Man, Rogosa and Sharpe (MRS)	Curiel et al. [40]
	*Lacticaseibacillus rhamnosus*	DSM 20021		Coman et al. [41]
	*Pediococcus pentosaceus*	DSM 20336		Ledesma et al. [42]
Yeast	*Meyerozyma guilliermondii*	BISA 2074	Yeast Extract, Peptone, Dextrose (YPD)	Martorell et al. [43]
	*Debaryomyces hansenii*	JCM 2162		Duarte et al. [44]
	*Zygosaccharomyces parabailii*	BISA 1307		Zuehlke et al. [45]
Pathogenic bacteria	*Salmonella typhimurium*	BISA 3969	Brain Heart Infusion (BHI)	N.A.
	*Listeria innocua*	BISA 3008		

**Table 2:** *G*′ and *G*″ at 1 Hz and plateau modulus values for YPEc and YPEn emulsions.

YPEc	*G*′ (Pa)	*G*″ (Pa)	$G_{N}^{0}$ (Pa)
Control	743.47 ± 45.5 ^b^	108.43 ± 6.9 ^c^	621.7 ± 59.2 ^b^
Red Cabbage FD	642.6 ± 5 ^b^	88.95 ± 4.5 ^d^	534.45 ± 3.2 ^b^
Red Cabbage Ext	636.07 ± 10.1 ^b^	86.22 ± 5.3 ^d^	515.43 ± 27 ^b^
ButterflyPeaC	1616.86 ± 45.7 ^a^	253.59 ± 8.6 ^b^	1343.92 ± 21.4 ^a^
ButterflyPeaExt	1771.32 ± 89.8 ^a^	314.57 ± 20.6 ^a^	1428.11 ± 71.1 ^a^
YPEn	*G*′ (Pa)	*G*″ (Pa)	$G_{N}^{0}$ (Pa)
Control	1147.67 ± 91.1 ^c^	171.63 ± 15.9 ^b,c^	780.1 ± 54 ^b^
Red Cabbage FD	1372 ± 36 ^b,c^	201.2 ± 4.7 ^b^	1082.5 ± 62.5 ^a,b^
Red Cabbage Ext	890.23 ± 69.1 ^d^	131.77 ± 5.7 ^c^	741.53 ± 54.7 ^b^
ButterflyPeaC	1582.51 ± 44.2 ^a^	241.52 ± 7.6 ^a^	1361.25 ± 64.9 ^a^
ButterflyPeaExt	1583.94 ± 111.3 ^a,b^	266.11 ± 15.4 ^a^	1321.73 ± 116.2 ^a^

Values are presented as mean ± standard deviation. Different superscript letters in each column represent statistical differences according to Tukey’s HSD test (*p* < 0.05).

**Table 3:** Zero shear rate limiting viscosity (*η*_0_) consistency coefficient (*k*) and deformation thinning rate (*n*) obtained from fitting experimental neutral and complete emulsions data to the modified Cross–Williamson’s model.

YPEc	*η*_0_ (10^3^ Pa·s)	*k* (10^1^ s)	*n* (Dimensionless)	r^2^
Control	11.28 ± 0.9 ^b^	15.09 ± 0.5 ^d^	0.035 ± 0.003 ^b,d^	0.9991
Red Cabbage FD	9.72 ± 0.1 ^b^	21.48 ± 0.1 ^b^	0.055 ± 0.055 ^c,d^	0.9984
Red Cabbage Ext	10.15 ± 0.3 ^b^	15.52 ± 0.2 ^d^	0.042 ± 0.005 ^a,b^	0.9997
ButterflyPeaC	24.14 ± 0.8 ^a^	19.95 ± 0.1 ^c^	0.045 ± 0.007 ^a,b^	0.9972
ButterflyPeaExt	22.45 ± 2.0 ^a^	23.36 ± 1.5 ^a^	0.042 ± 0.002 ^a,c^	0.9893
YPEn	*η*_0_ (10^3^ Pa∙s)	*k* (10^1^ s)	*n* (dimensionless)	r^2^
Control	16.99 ± 0.9 ^b^	10.78 ± 0.3 ^c^	0.004 ± 0.001 ^b^	0.9991
Red Cabbage FD	14.53 ± 2.0 ^a,b^	10.08 ± 0.8 ^c^	0.004 ± 0.001 ^b^	0.9968
Red Cabbage Ext	14.68 ± 1.2 ^b^	10.96 ± 1.5 ^c^	0.02 ± 0.009 ^b^	0.9975
ButterflyPeaC	24.65 ± 0.5 ^a^	22.93 ± 0.4 ^b^	0.052 ± 0.006 ^a^	0.9954
ButterflyPeaExt	17.38 ± 0.3 ^b^	45.37 ± 0.1 ^a^	0.035 ± 0.001 ^a^	0.9738

Values are presented as mean ± standard deviation. Different superscript letters in each column represent statistical differences according to Tukey’s HSD test (*p* < 0.05).

**Table 4:** CIELab and pH measurements from emulsions produced with both YPEc and YPEn and red cabbage powder (RCFD), red cabbage extract (RCExt), butterfly pea commercial extract (BPc) and butterfly pea extract (BPExt).

YPEc	pH	L*	a*	b*	∆*E*
Control	4.09 ± 0.04 ^a,b^	78.84 ± 0.29	−1.60 ± 0.2	11.55 ± 0.83	16.22 ± 0.68
Red Cabbage FD	4.26 ± 0.02 ^a^	75.68 ± 0.03	4.08 ± 0.64	8.52 ± 0.17	10.08 ± 0.24
Red Cabbage Ext	4.28 ± 0.01 ^a^	42.24 ± 1.88	44.15 ± 0.86	−12.42 ± 0.45	65.70 ± 1.70
ButterflyPeaC	4.11 ± 0.01 ^b^	70.37 ± 0.53	1.82 ± 0.19	3.05 ± 0.08	16.03 ± 0.28
ButterflyPeaExt	4.08 ± 0.01 ^b^	50.80 ± 0.58	13.17 ± 0.29	−11.64 ± 0.23	42.19 ± 0.64
YPEn	pH	L*	a*	b*	∆*E*
Control	4.08 ± 0.1 ^a,b^	83.34 ± 0.53	−2.3 ± 0.02	8.18 ± 0.33	15.11 ± 0.58
Red Cabbage FD	4.06 ± 0.02 ^b^	78.63 ± 0.52	6.57 ± 0.24	4.33 ± 0.14	14.74 ± 0.22
Red Cabbage Ext	4.09 ± 0.01 ^b^	42.87 ± 2.46	44.4 ± 1.23	−6.67 ± 0.72	66.64 ± 2.69
ButterflyPeaC	4.11 ± 0.01 ^b^	74.07 ± 0.20	2.11 ± 0.40	−3.31 ± 0.42	21.08 ± 0.53
ButterflyPeaExt	4.11 ± 0.01 ^b^	54.74 ± 0.50	13.75 ± 0.3	−15.1 ± 0.27	44.59 ± 0.60

Values are presented as mean ± standard deviation. Different superscript letters in each column represent statistical differences according to Tukey’s HSD test (*p* < 0.05).

**Table 5:** Visual aspect of the emulsions produced with both complete and neutral YPE and red cabbage powder (RCFD), red cabbage extract (RCExt), butterfly pea commercial extract (BPc) and butterfly pea extract (BPExt).

	Control	RCFD	RCExt	BPc	BPExt
YPEc	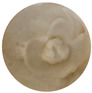	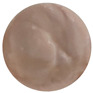	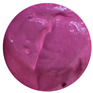	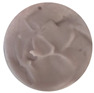	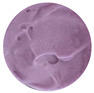
YPEn	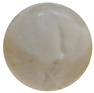	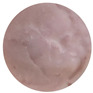	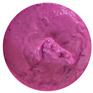	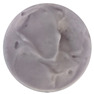	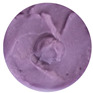

## Data Availability

Further information will be made available upon request to the corresponding author.

## Supplementary Materials

The following supporting information can be downloaded at https://www.mdpi.com/article/10.3390/antiox15030351/s1, Figure S1: HPLC chromatograms of (a) Red cabbage extract and (b) butterfly pea flower extract. Peak numbers correspond to the respective tentative identifications in Tables S1 and S2; Figure S2: Plated 10-fold dilutions of Complete (a and c) and Neutral (b and d) emulsions inoculated with yeasts (MG—*Meyerozyma guilliermondii*, DH—*Debaryomyces hansenii*, and ZP—*Zygosaccharomyces parabailii*), lactic acid bacteria (LB—*Levilactobacillus brevis*, LcR—*Lacticaseibacillus rhamnosus* and PP—*Pediococcus pentosaceus*). Each cut-out represents a registered timepoint, one example of each replica is shown. Table S1: Tentative Identification of the main phenolic compounds present in red cabbage extract by LC-MS where RT is Retention time, UV max. is the maximum absorption wavelength, [M]^+^ is the ion mass, MS^2^ is the fragmentation pattern. Identifications were attributed based on previous studies by Pereira et al. and Silva et al. [28,29]; Table S2: Tentative Identification of the main phenolic compounds present in red cabbage extract by LC-MS where RT is Retention time, UV max. is the maximum absorption wavelength, [M]^+^ is the ion mass, MS^2^ is the fragmentation pattern. Identifications were attributed based on previous studies by Pereira et al. and Silva et al. [28,29].

## Abbreviations

The following abbreviations are used in this manuscript:

YPE	Yeast protein extract
EDTA	Ethylenediaminetetraacetic acid
YPEc	Complete yeast protein extract
YPEn	Neutral yeast protein extract
DSC	Differential Scanning calorimetry
SAOS	Small amplitude oscillatory shear
PV	Peroxide value
MRS	De Man, Rogosa and Sharpe
YPD	Yeast extract, peptone, dextrose
BHI	Brain heart infusion
RCFD	Red cabbage freeze-dried
BPc	Butterfly pea flower powder
RCExt	Red cabbage extract
BPExt	Butterfly pea flower extract
DH	*Debaryomyces hansenii*
LcR	*Lacticaseibacillus rhamnosus*
LIS	*Listeria innocua*
SAL	*Salmonella typhimurium*
