# Supplementary material for: Yeast Protein Extract Emulsions Supplemented with Polyphenolic Compounds: Physical, Chemical and Stability Properties of Colorful Emulsions
Source: Antioxidants (Basel). 2026 Mar 11;15(3):351. doi: 10.3390/antiox15030351 (PMC13024442; doi:10.3390/antiox15030351)
Supplement: Supplementary file 1 [file antioxidants-15-00351-s001.zip › antioxidants-4168145-SI.pdf]

Supporting Information

Yeast protein extract emulsions supplemented with polyphenolic compounds: physical, chemical and stability properties of colourful emulsions

Bernardo Almeida[1], Ana Catarina Costa[2], Filipe Vinagre[2], Catarina Prista[2], Filipe Centeno[3], Victor de Freitas[1], Anabela Raymundo[2], Susana Soares[1]

[1]REQUIMTE, LAQV, Department of Chemistry and Biochemistry, Faculty of Sciences, University of Porto, Rua do Campo Alegre, s/n, 4169-007, Porto, Portugal

[2]LEAF – Linking Landscape, Environment, Agriculture and Food Research Center, Associated Laboratory TERRA, Superior Agronomy Institute of the University of Lisbon, Tapada da Ajuda, 1349-017, Lisbon, Portugal

[3]Proenol, SA, Travessa das Lages 267, 4410-308, Canelas, Portugal

Corresponding author: susana.soares@fc.up.pt

Polyphenol extraction and quantification

Fresh red cabbage (*Brassica oleracea var. capitata f. rubra*) was bought from a local market, cut into small pieces and frozen, freeze-dried, made into a powder, and kept at -18°C until extraction (RCFD). Butterfly pea flower (*Clitoria ternatea*) (BPC) produced by Zhejiang Binmei Biotechnology CO. LTD was used as is for the extraction. Red cabbage extract (RCExt) was produced by adding RCFD to boiling water (1:20 w/v) and the extraction was carried out without further heating, under agitation, for 30 minutes. After 30 minutes, solvent was exchanged and this process was repeated another 2 times until no further color was extracted. This aqueous extract was then purified by C18 gel purification Lichroprep® RP-18 (PSz 40 to 63 μm), using deionized water to fully remove sugars and eluted with acidifed methanol. Methanolic extract was then dried using a rotary evaporator, resolubilized in water and freeze-dried. Butterfly pea extract (BPExt) was produced by adding BPC to room temperature deionized water (1:20 w/v) until total dissolution and purified using the same method as RCExt.

Extracts were analysed on a Vanquish HPLC (Thermo Fischer Scientific) equipped with an Agilent Poroshell 120, (RP C18, 250 × 4.6mm, 2.7 μm particle size) and mass spectrometry was conducted on a Thermo Scientific Vanquish UPLC equipped with a linear ion trap mass spectrometer (LTQ XL™) using ESI source in positive ion mode. Separation was conducted using Solvent A: 1% formic acid in water and solvent B: 1% formic acid in 30% acetonitrile and 70% water. Gradient elution begins with 35% solvent B, increasing 80% until 65 minutes; at 66 minutes solvent B increases to 100% and decreasing to 35% after 76 minutes; gradient returns to initial conditions until the 85-minute mark. Flow rate used for each analysis was set to 0.4 ml·ml-1 with detection at 280 nm, 340 nm and 540 nm [1]. For quantification, Cianidin-3-glucoside and pure Ternatin D1 were used as standards for a calibration curve for Red Cabbage and Butterfly Pea

Flower respectively, resulting in a Cianidin-3-glucoside curve (Area = 22454.67 [Anthocyanin concentration mg/ml] -1.924 , r^2 = 0.9916 and a Ternatin D1 curve Area = 283.96 [Anthocyanin concentration mg/ml] + 5.2906, r^2= 0.99. As such, anthocyanin concentration was calculated to be 91.54% and ternatin concentration was calculated to be 87.95%.

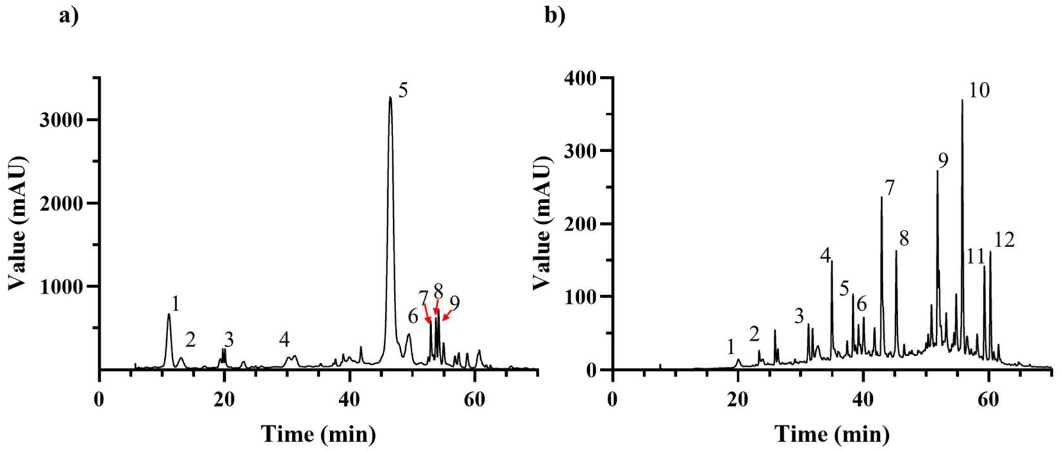

Figure S1. HPLC chromatograms of a) Red cabbage extract and b) butterfly pea flower extract. Peak numbers correspond to the respective tentative identifications in table S1 and S2.

Table S1- Tentative Identification of the main phenolic compounds present in red cabbage extract by LC-MS where RT is Retention time, UV max. is the maximum absorption wavelength, [M]+ is the ion mass, MS^2 is the fragmentation pattern. Identifications were attributed based on previous studies by Pereira et al. and Silva et al. [1,2].

Peak	RT (min)	UV max. (nm)	$[M]^+$ (*m/z*)	MS^2 (*m/z*)	Tentative Identification
1	11,83	513	773	611; 449; 286	cyanidin-3-diglucoside-5-glucoside
2	13,59	513	611	449; 286	cyanidin-3,5-diglucoside
3	20,34	523	1141	979; 449	cyanidin-3-(sinapoyl)-triglucoside-5-glucoside
4	29,91	522	1081	919	Cyanidin-3-(caffeoyl)(p-coumaroyl)-diglucosides-5-glucoside
5	45,96	523	919	757; 449; 286	cyanidin-3-(p-coumaroyl)-diglucoside-5-glucoside
6	48,85	523	817	449,287	Cyanidin-3-(sinapoyl)glucoside-5-glucoside
7	52,31	532	1125	963; 736; 449	cyanidin-3-(feruloyl)(feruloyl)-diglucoside-5-glucoside
8	53,02	535	1155	993; 449	cyanidin-3-(feruloyl)(sinapoyl)-diglucoside-5-glucoside
9	53,75	535	1185	1023; 440	cyanidin-3-(sinapoyl)-(sinapoyl)-diglucoside-5-glucoside

Table S2 -Tentative Identification of the main phenolic compounds present in red cabbage extract by LC-MS where RT is Retention time, UV max. is the maximum absorption wavelength, [M]+ is the ion mass, MS^2 is the fragmentation pattern. Identifications were attributed based on previous studies by Pereira et al. and Silva et al. [1,2].

Peak	RT (min)	UV max. (nm)	$[M]^+$ (*m/z*)	MS^2 (*m/z*)	Tentative Identification
1	20,21	538	1405	1243; 1081	Ternatin A3
2	23,59	541	1491	1243; 1081	Ternatin C2
3	32,29	541	1329	1081;1021	Ternatin B4
4	35,95	549	1799	1551; 1081	Ternatin A2
5	37,13	255	757	611,465	Quercetin-3-O-(2''-alpha-L-rhamnosyl)-rutinoside
6	40,05	541	1167	919; 611	Ternatin D3
7	42,14	265	594	449; 287	Kaempferol-3-O-neohesperidoside
8	45,3	549	1475	1227; 1167	Ternatin D2
9	51,7	547	1637	1593; 1389	Ternatin B3
10	55,92	537	1329	1167; 919	Ternatin C1
11	59,31	555	1783	1637; 1475	Ternatin D1
12	60,25	548	1946	1697; 1535	Ternatin B1

Yeast protein extract quantification

Protein content of each yeast protein extract was determined according to the Kjedahl method [3]. 1g of each YPE, 6.7 g of copper sulphate, 0.7 g of potassium sulphate and 10 ml of sulfuric acid were added to pyrolysis tubes. Blank samples were performed the same way, with 10ml of water, to obtain background nitrogen. Samples were digested in a VELP Scientific DK6 for 60 minutes at 420°C. Following digestion, samples were steam distilled in a VELP Scientifica UDK 129 distillation unit with 35% (w/v) sodium hydroxide, and the resulting steam being captured in a 4% (w/v) boric acid solution. Total nitrogen is then titrated with standardized hydrochloric acid at 1 mol·L^{-1}. % nitrogen was then multiplied by a factor 6.25 to obtain final protein concentration. Final yeast protein concentration were calculated as 70.55% and 56.25% for YPEn and YPEc respectively.

Anti-microbial assays

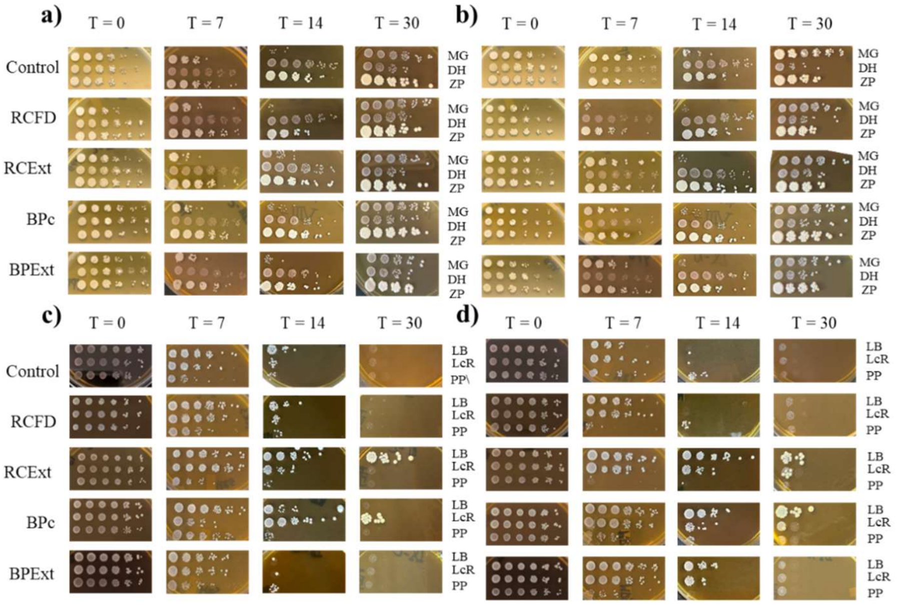

Figure S2. Plated 10-fold dilutions of Complete (a, and c) and Neutral (b and d) emulsions inoculated with yeasts (MG- *Meyerozyma guilliermondii*, DH – *Debaryomyces hansenii*, and ZP- *Zygosaccharomyces parabailii*), lactic acid bacteria (LB- *Levilactobacillus brevis*, LcR – *Lacticaseibacillus rhamnosus* and PP- *Pediococcus pentosaceus*). Each cut-out represents a registered timepoint, one example of each replica is shown.

1. Silva, I.E.; Vieira, J.; Guerreiro, C.; Oliveira, J.; Brandão, E.; de Freitas, V.; Soares, S. Molecular Insights into the Astringency of Clitoria ternatea Tea: Role of Phenolic Structure, Oral Constituents, and pH. *Journal of Agricultural and Food Chemistry* **2025**, *73*, 26957-26971.
2. Pereira, A.R.; Fernandes, V.C.; Delerue-Matos, C.; de Freitas, V.; Mateus, N.; Oliveira, J. Exploring acylated anthocyanin-based extracts as a natural alternative to synthetic food dyes: Stability and application insights. *Food Chemistry* **2024**, *461*, 140945.
3. Bradstreet, R.B. Kjeldahl method for organic nitrogen. *Analytical Chemistry* **1954**, *26*, 185-187.
